# Lipid Peroxidation and Chlorophyll Fluorescence of Photosystem II Performance during Drought and Heat Stress is Associated with the Antioxidant Capacities of C3 Sunflower and C4 Maize Varieties

**DOI:** 10.3390/ijms21144846

**Published:** 2020-07-09

**Authors:** Dilek Killi, Antonio Raschi, Filippo Bussotti

**Affiliations:** 1 Department of Agrifood Production and Environmental Sciences (DiSPAA), Piazalle della Cascine 18, 50144 Florence, Italy; filippo.bussotti@unifi.it; 2 The National Research Council of Italy, Institute of BioEconomy (CNR–IBE), Via Giovanni Caproni 8, 50145 Florence, Italy; antonio.raschi@ibe.cnr.it

**Keywords:** phenotyping, plant antioxidant enzymes, glutathione reductase, superoxide dismutase, catalase, peroxidase, ascorbate peroxidase, food security

## Abstract

Agricultural production is predicted to be adversely affected by an increase in drought and heatwaves. Drought and heat damage cellular membranes, such as the thylakoid membranes where photosystem II occurs (PSII). We investigated the chlorophyll fluorescence (*ChlF*) of PSII, photosynthetic pigments, membrane damage, and the activity of protective antioxidants in drought-tolerant and -sensitive varieties of C3 sunflower and C4 maize grown at 20/25 and 30/35 °C. Drought-tolerant varieties retained PSII electron transport at lower levels of water availability at both temperatures. Drought and heat stress, in combination and isolation, had a more pronounced effect on the *ChlF* of the C3 species. For phenotyping, the maximum fluorescence was the most effective *ChlF* measure in characterizing varietal variation in the response of both species to drought and heat. The drought-tolerant sunflower and maize showed lower lipid peroxidation under drought and heat stress. The greater retention of PSII function in the drought-tolerant sunflower and maize at higher temperatures was associated with an increase in the activities of antioxidants (glutathione reductase, superoxide dismutase, catalase, peroxidase, and ascorbate peroxidase), whereas antioxidant activity declined in the drought-sensitive varieties. Antioxidant activity should play a key role in the development of drought- and heat-tolerant crops for future food security.

## 1. Introduction

As average global temperatures rise, the frequency of drought events and heatwaves will increase [1,2,3]. This combination of drought and heat stress will have significant negative implications for agricultural production of both C3 [4,5] and C4 [6] crops. The effects of drought [7,8] and higher temperatures [9,10] on crop plants have been studied widely in isolation, but their potential combined impact is less clearly defined. To ensure food security, it is necessary to identify traits that confer tolerance to drought and heat stress towards developing more tolerant varieties [11,12,13]. Despite the frequent occurrence of drought events with heatwaves [1,3], the genetic pathways regulating plant responses to drought and heat stress are largely distinct [13]. Analysis of the physiological mechanisms involved in crop responses to drought and heat stress may elucidate the photosynthetic and protective behaviors that underpin tolerance to these stresses. Chlorophyll fluorescence (*ChlF*) allows rapid non-destructive collection of data relating to the performance of photosystem II (PSII) [14] and is highly sensitive to the deleterious effects of drought [15,16] and heat [17,18] stress. To protect and stabilize the thylakoid membrane where PSII electron transport occurs, plants possess protective antioxidant mechanisms [19,20]. Indeed, the stability of cellular membranes is a key determinant of plant tolerance to heat and drought stress [21].

As the availability of water for uptake by plants declines, stomatal pores close to reduce water-loss as a result of transpiration, and to prevent desiccation [7,22,23]. A decrease in stomatal conductance (*G*_s_) causes lower availability of CO_2_ for photosynthesis (*P*_N_). This results in a decrease in the use of energy for photochemistry, and necessitates an increase in the dissipation of energy as heat to prevent damage to the photosynthetic apparatus [14,24]. Reduced photochemical energy usage can also result in increased generation of reactive oxygen species (ROS) [19,25]. In some species, during the initial stages of drought stress, the activity of antioxidant systems increases to protect against excessive levels of ROS [20]. During prolonged or severe drought the capacity of plants to dissipate excess energy or neutralize harmful ROS may become overwhelmed, leading to damage to the structure and function of cellular membranes; in particular, the thylakoid membranes within the chloroplast where PSII electron transport takes place [15,19,24]. The thylakoid membranes are also vulnerable to heat stress [17,26,27,28,29]. Heat stress also induces a decrease in *P*_N_ by reducing the affinity of ribulose-1,5-bisphosphate carboxylase/oxygenase (RubisCO) for CO_2_ [30] and the activity of RubisCO activase [9,26], possibly reducing the capacity for photosystem I (PSI) to act as an electron receiver for PSII [16,31], thus impairing the use of energy for photochemistry through PSII [14]. As such, the thylakoid membranes are particularly sensitive to the effects of both drought and heat stress, and any disruption to the structure and function of the thylakoid membranes is evident in reductions in the efficiency of electron transport during PSII [17,26,32]. Chlorophyll fluorescence measures of PSII performance should therefore provide a quantitative measure of the impact of drought and heat stress on the functionality of thylakoid membranes. Moreover, comparison of different *ChlF* parameters may indicate the most sensitive or diagnostic measure(s) for use in rapid phenotyping for drought and heat tolerance using active *ChlF* methods [33,34].

During drought stress, the lipids that constitute cellular membranes become damaged via peroxidation [35]. Plant species with increased tolerance to drought stress exhibit greater antioxidant activity than species that are more susceptible to drought [36]. Heat stress also increases the production of ROS and peroxidation of lipid membranes [37,38,39]. Varieties of wheat (*Triticum aestivum* L.) [40] and cotton (*Gossypium hirsutum* L.) [41] with enhanced heat tolerance also exhibit significantly higher levels of antioxidant activity than their less tolerant counterparts. A combination of drought and heat stress increases the peroxidation of membrane lipids more rapidly than both stresses in isolation in Kentucky bluegrass (*Poa pratensis* L.) and tall fescue (*Festuca arundinacea* Schreb). This membrane degradation coincided with reductions in antioxidant activity, suggesting that the protective mechanisms functioning to stabilize plant membranes were overwhelmed when drought and heat stress were combined [35]. The capacity of antioxidant mechanisms to protect plant physiology and membranes likely plays an integral role in determining crop responses to drought and heat [41]. Analysis of the activity of major antioxidants alongside *ChlF* and lipid peroxidation should provide insights into the functioning of photosynthetic thylakoid membranes and the protective mechanisms that support the maintenance of *P*_N_ during episodes of high oxidative stress.

This study builds upon previous work where the impact of drought and heat stress on the leaf gas exchange and morphological characteristics of the same drought-tolerant and -sensitive C3 sunflower (*Helianthus annuus* L.) and C4 (*Zea mays* L.) maize were examined [10]. The drought-tolerant and -sensitive varieties of sunflower and maize exhibited contrasting leaf gas exchange and growth responses to drought when grown at 25 and 35 °C. Increased temperature had little effect on *P*_N_ in the maize varieties due to C4 photosynthesis (accumulation of oxaloacetic acid within the bundle sheath effectively concentrating CO_2_ at the RubisCO carboxylation site), thus eliminating the enhanced photorespiration at 35 °C that negatively affected sunflower [17]. The drought-tolerant sunflower sustained greater *P*_N_ at 35 °C by retaining RubisCO activity [42]. We intend to explore the impact of drought and heat stress, in combination and isolation, on the functionality of PSII and the protective mechanisms that stabilize the photosynthetic membranes where electron transport occurs. We hypothesize that the more drought-tolerant varieties of sunflower and maize will possess enhanced antioxidant activities [35,36,41] and will retain PSII performance to a greater extent under drought and heat stress. This study aims to: (i) Investigate the impact of drought and heat stress on *ChlF* parameters of C3 sunflower and C4 maize as an indicator of PSII performance; (ii) identify the most effective *ChlF* measures for the characterization of drought and heat stress, and identify tolerant varieties for phenotyping using active *ChlF*; (iii) examine differences in antioxidant capacity and responses to drought and heat stress in the varieties of sunflower and maize; and (iv) explore whether the capacity of protective antioxidant systems are linked to the maintenance of PSII under drought and heat stress, and whether these attributes are desirable in the selection and development of crop varieties to maintain productivity in regions predicted to be affected by an increased frequency of drought events and heatwaves.

## 2. Results

### 2.1. Leaf Gas Exchange

**Figure 1:**
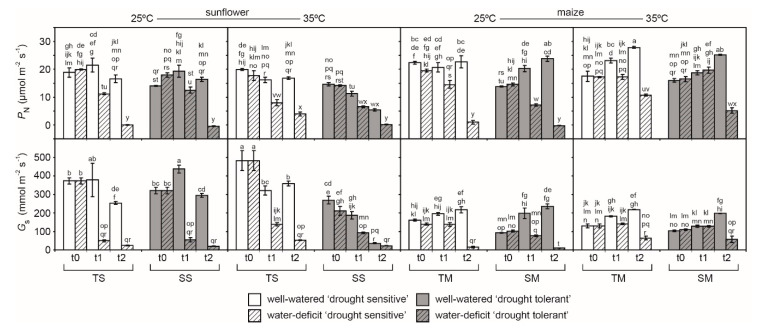
Photosynthesis (*P*_N_) and stomatal conductance (*G*_s_) measured using gas exchange of drought-tolerant (white) and -sensitive (gray) varieties of sunflower and maize under well-watered (open) and drought (hatched) conditions at 25 and 35 °C. Gas exchange measurements represent the average of values of four replicate plants collected at the three sampling points (t0, t1, t2). TS = drought-tolerant sunflower; SS = drought-sensitive sunflower; TM = drought-tolerant maize; SM = drought-sensitive maize. Error bars indicate one standard error either side of the mean. Letters indicate homogenous groups determined using a one-way ANOVA and LSD post-hoc test.

Stomatal conductance values of well-watered C3 sunflower were generally higher than those of the C4 maize. Rates of *P*_N_ were identical at t0 at both 25 and 35 °C in the drought-tolerant varieties of both species. As soil dried, *G*_s_ declined at t1 and t2 in the C3 sunflower, with more rapid reductions in drought-sensitive sunflower (SS) at the higher temperature. Stomatal conductance was generally lower in the 35 °C treatment in both TS and SS. The effect of temperature on *G*_s_ was less apparent in both maize varieties. The influence of temperature on *P*_N_ and *G*_s_ as soil dried was less apparent on the tolerant and sensitive maize than the sunflower (Figure 1).

### 2.2. Chlorophyll Fluorescence Parameters

**Figure 2:**
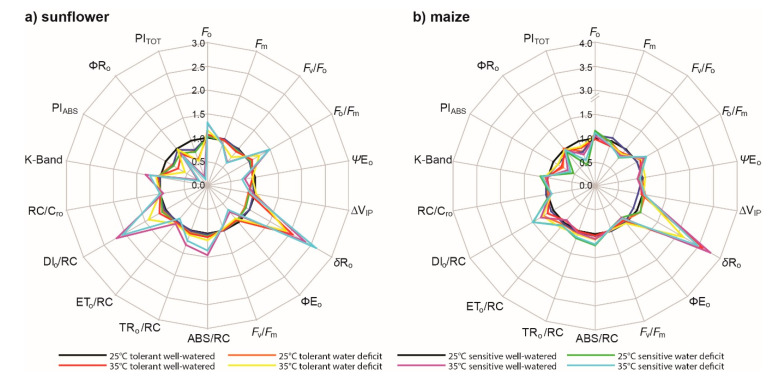
Spider plot of *ChlF* parameters of sunflower (**a**) and maize (**b**) at t3 (see Materials and Methods for definitions and descriptions) extrapolated from the OJIP transient expressed in relation to values of the tolerant variety receiving full irrigation in the 25 °C treatment. Definitions for parameters can be found in Supplementary Data Table S1.

A summary of the *ChlF* parameters in the final two days of the drought treatment and their relationship to the values of well-watered plants grown at 25 °C assessed using a non-parametric test are provided in Table 1 and relative changes in Figure 2. The effects of drought and heat stress were more apparent in the *ChlF* characteristics of the C3 sunflower varieties. Total photosynthetic index (PI_tot_), IP-phase, and K-band did not effectively differentiate the effects of drought stress on sunflower at 25 °C and drought on maize at both temperature treatments. The maximum quantum yield of PSII (*F*_v_/*F*_m_) was largely insensitive (although not universally) to the impact of drought stress in the 25 °C treatment. However, at the higher temperature significant reductions in the maximum quantum yield were apparent in both species. The fluorescence maximum (*F_m_*) was the most sensitive parameter in both sunflower and maize varieties in terms of drought treatment and varietal effects at both temperatures.

**Figure 3:**
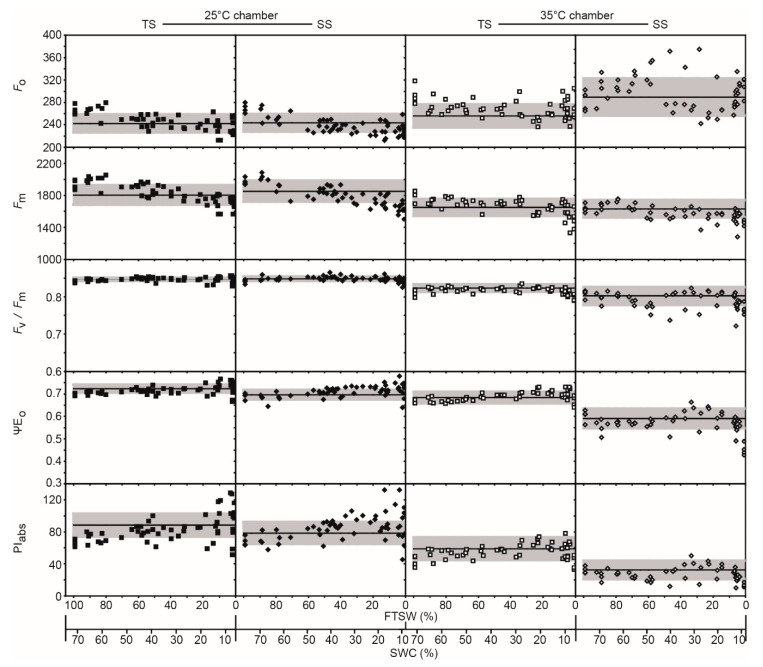
The response of *ChlF* parameters in drought-tolerant (TS) (square symbols) and -sensitive (SS) (diamond symbols) varieties of sunflower to soil drying at 25 °C (solid symbols) and 35 °C (open symbols). Data are plotted against the fraction of transpirable soil water (FTSW) and a calibration between FTSW and soil water content (SWC). The solid horizontal line indicates the mean value of the well-watered control plants (*n* = 4) during the experimental period and the gray shading indicates ± standard deviation either side of the mean. The complete FTSW response of *ChlF* parameters can be found in Supplementary Data Figure S1.

Growth at 35 °C induced an increase in *F*_o_ and decrease in *F*_m_ values of both sunflower varieties under well-watered conditions (Figure 3). At 25 °C no difference was observed in the *F*_v_/*F*_m_ values of the drought-tolerant (TS) and -sensitive (SS) sunflower; however, at 35 °C *F_v_/F_m_* values declined to a greater extent in the SS. Values of ΨE_o_ and PI_(abs)_ allowed differentiation between tolerant and sensitive varieties at 25 °C, and these differences were more pronounced at 35 °C. The *ChlF* parameters ΨE_o_, RC/ABS, PI_abs_, IP-phase, and K-band derived from the OJIP curve did not characterize the drought kinetics of the sunflower varieties at either temperature. Rather, as water availability declined towards lower fraction of transpirable soil water (FTSW) values, these *ChlF* parameters became more variable. The *F*_m_ was effective in demonstrating reduced photochemical energy usage at lower FTSW values in both TS and SS (Figure 2a or Figure 3, Table 1).

The minimal fluorescence values of well-watered maize were not as strongly affected by temperature as was the sunflower (Figure 2b and Table 1). Nevertheless, *F*_m_ of maize was sensitive to growth at 25 and 35 °C. The *ChlF* parameters ΨE_o_, PI_abs_, and PI_tot_ all showed non-significant declines (Table 1) at the higher temperature in the well-watered maize varieties. The kinetics of drought stress were apparent in *F*_o_, *F*_m_, *F*_v_/*F*_m_, and PI_abs_ values of maize at 25 but not 35 °C.

**Figure 4:**
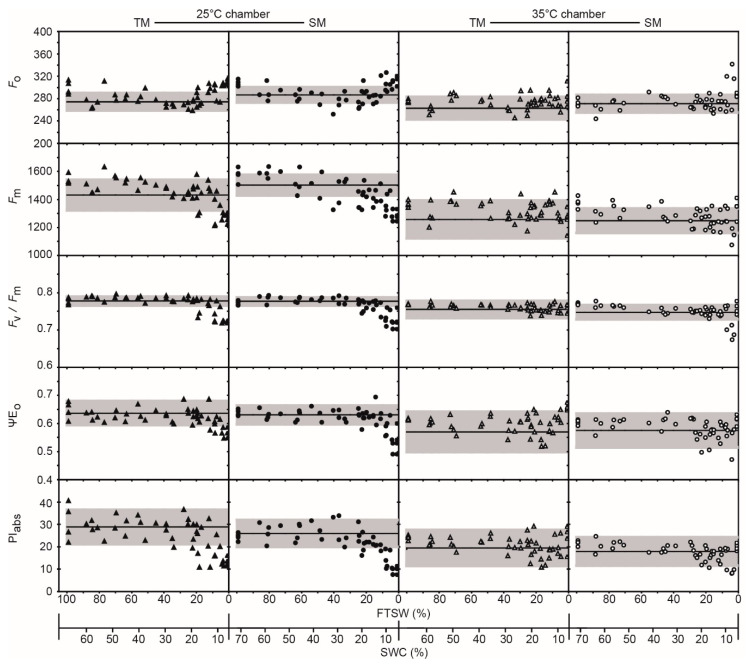
The response of *ChlF* parameters in drought-tolerant (TM) (triangle symbols) and -sensitive (SM) (circle symbols) varieties of maize to soil drying at 25 (solid symbols) and 35 °C (open symbols). Data are plotted against the FTSW and a calibration between FTSW and SWC. The solid horizontal line indicates the mean value of the well-watered control plants (*n* = 4) during the experimental period and the gray shading indicates ± standard deviation either side of the mean. The complete FTSW response of *ChlF* parameters can be found in Supplementary Data Figure S2.

**Figure 5:**
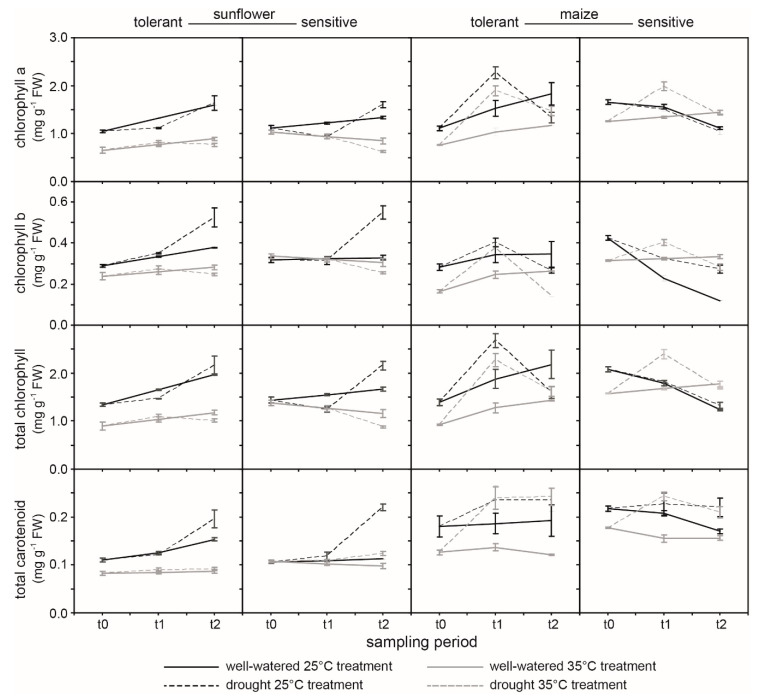
Chlorophyll a, chlorophyll b, total chlorophyll, and total carotenoid content in drought-tolerant and -sensitive varieties of sunflower and maize exposed to soil drying at 25 and 35 °C at three sampling points (t0, t1, t2). The solid unbroken line indicates well-watered control plants, and the dashed line indicates drought plants. The black line indicates the 25 °C treatment, and the gray line indicates the 35 °C treatment. Differences in homogenous groups for each species (maize and sunflower separately) were determined using a one-way ANOVA and LSD post-hoc test, which are provided in supplementary data (see Supplementary Data Table S2).

Lower PI_abs_ in sunflower (Figure 3) and maize (Figure 4) when grown at 35 °C coincided with reduced levels of photosynthetic pigments (Figure 5). Foliar levels of chlorophyll a, total chlorophyll, and total carotenoids were lower in plants grown at the higher temperature. The TS generally possessed greater levels of photosynthetic pigments than its sensitive counterpart. In contrast, at t0 the drought-sensitive maize (SM) exhibited higher levels of chlorophyll a, chlorophyll b, total chlorophyll, and total carotenoids, but these declined over the course of the experiment in plants grown at 25 °C, while pigment concentrations generally increased in the drought-tolerant maize (TM) (Figure 5).

### 2.3. Antioxidant Enzyme Activities

**Figure 6:**
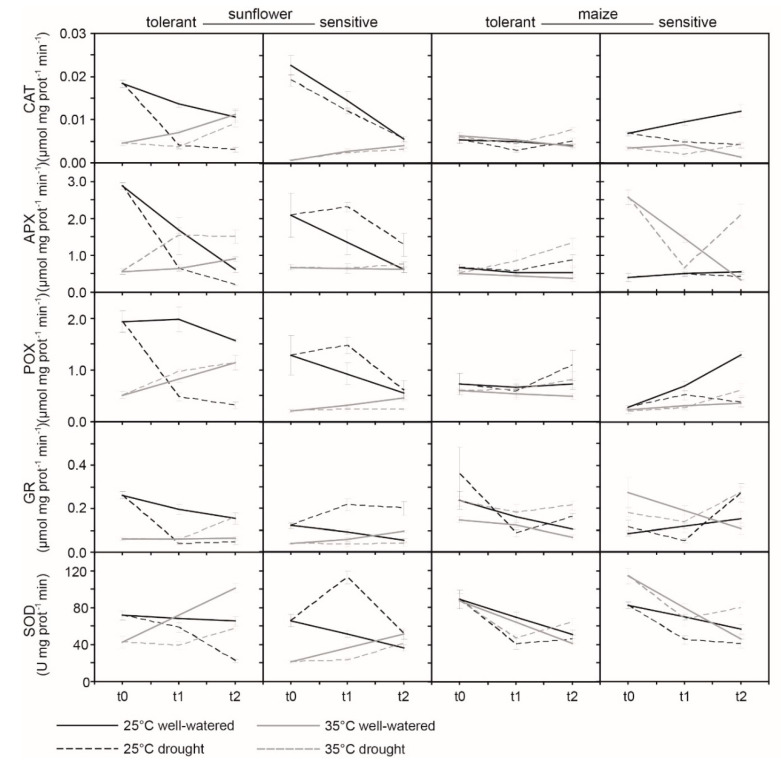
Antioxidant enzyme (catalase (CAT), ascorbate peroxidase (APX), peroxidase (POX), glutathione reductase (GR)) activities in drought-tolerant and -sensitive varieties of sunflower and maize exposed to soil drying at 25 and 35 °C at three sampling points (t0, t1, t2). Line indications are the same as in Figure 5. Differences in homogenous groups for each species (maize and sunflower separately) determined using a one-way ANOVA and LSD post-hoc test are provided in supplementary data (see Supplementary Data Table S2).

**Figure 7:**
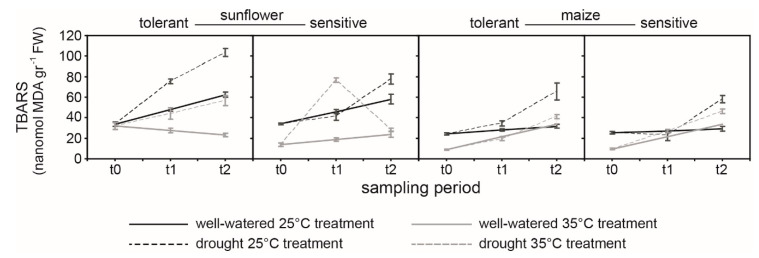
Lipid peroxidation level (thiobarbituric acid reactive substances (TBARS) content) in drought-tolerant and -sensitive varieties of sunflower and maize exposed to soil drying at 25 and 35 °C at three sampling points (t0, t1, t2). Line indications are the same as in Figure 5. Differences in homogenous groups for each species (maize and sunflower separately) determined using a one-way ANOVA and LSD post-hoc test are provided in supplementary data (see Supplementary Data Table S2).

At t0, levels of all antioxidants were lower in TS and SS varieties grown at 35 °C (Figure 6). This coincided with greater malondialdehyde (MDA) levels in the sunflower varieties from the lower temperature treatment (Figure 6). Levels of antioxidants declined in well-watered sunflower varieties over the course of the study at 25 °C. Drought stress induced more rapid reductions in antioxidant levels in TS at 25 °C, but increases in superoxide dismutase (SOD), ascorbate peroxidase (APX), peroxidase (POX), and glutathione reductase (GR) activity in the SS (Figure 6). Both TS and SS exhibited similar levels of lipid peroxidation during drought stress at 25 °C (Figure 7). At 35 °C, the TS exhibited more pronounced increases in catalase (CAT), APX, POX, and SOD activities under both well-watered and drought conditions. Drought stress induced increased levels of thiobarbituric acid reactive substances (TBARS) indicative of membrane damage in both sunflower varieties; however, the increase at 35 °C was lower in TS than SS (Figure 7).

The maize varieties exhibited lower levels of antioxidant activity than that of the sunflower varieties at the start of the experimental period (Figure 6). At 25 °C the well-watered TM exhibited no change or declines in antioxidant levels over the course of the experiment, whereas the SM showed increases in CAT, POX, and GR levels (Figure 6). Over the duration of the study, both TM and SM exhibited increased lipid peroxidation under well-watered conditions. The TM variety largely showed no difference in the significance or direction (i.e., elevated, decreased, or maintained) of antioxidant response between plants grown at 25 and 35 °C (Figure 6). In contrast, SM showed reduced levels of CAT and POX activity and increased activities of APX, GR, and SOD at the higher temperature. Drought induced increased CAT, APX, POX, and GR activities in TM at 25 and 35 °C, but SM showed reduced CAT, POX, and SOD at 25 °C during the drought stress. At 35 °C, the SM when subject to drought showed increased CAT, APX, POX, GR, and SOD activities at t2. While the impact of temperature on the response of antioxidants was less pronounced between TM and SM varieties at 35 °C than between the contrasting sunflower varieties (Figure 6), it is noteworthy that drought induced an increase in activities of CAT, APX, POX, and GR at both temperatures, whereas such a consistent up-regulation of antioxidant activities during drought stress was not present in SM, indicative of varietal differences in maize. The impact of temperature, drought, and variety on the level of antioxidant activity was more apparent in sunflower than in maize—a three-way ANOVA was used to assess these interactions and the results are summarized in Table 2.

## 3. Discussion

Drought and heat stress frequently occur together in agricultural systems, causing impaired plant growth and loss of productivity. Both drought and heat stress disrupt the structure and function of plant membranes, such as the thylakoid membranes within the chloroplast where PSII takes place. To stabilize and protect these membranes from lipid peroxidation, plants possess antioxidant systems to neutralize ROS that are produced by an imbalance of energy usage experienced by plants during drought and/or heat stress. This study has shown contrasting *ChlF*, lipid peroxidation, and antioxidant responses to drought and/or heat stress in drought-tolerant and -sensitive varieties of C3 sunflower and C4 maize. The impact of drought and heat stress was more pronounced in sunflower and less evident in maize; consistent with the leaf gas exchange (Figure 1). Nonetheless, antioxidants also played a significant role in determining the PSII response of C4 maize to drought stress.

### 3.1. Chlorophyll Fluorescence Analysis of the Effects of Drought and Heat Stress

The maximum quantum yield of PSII is relatively stable under drought, only declining in the most severe stages of stress [14,43,44,45]. A decline in *F*_v_/*F*_m_ values as soil dried was not observed in sunflower, and only occurred at the lowest FTSW levels in maize at 25 °C (Figure 2, Figure 3 and Figure 4). Reduced PI_ABS_ and PI_TOT_ (Figure 2) indicate lower photochemistry in both sunflower and maize under drought and heat stress, consistent with leaf gas exchange measurements of *P*_N_ (Figure 1) [16,46]. Analysis of OJIP transients (JIP test) indicates that drought often results in a depression of the IP phase in crop species [33]; however, the IP phase of the sunflower and maize varieties was relatively insensitive to drought at both temperatures (Table 1; Figure 3 and Figure 4). An increase in *F*_o_ indicative of irreversible damage to PSII, decline of *F*_m_, and subsequent reduction of *F*_v_/*F*_m_ [47] was observed in maize at both temperatures but more pronounced in the SM variety; consistent with observations of increased lipid peroxidation damage (Figure 7) symptomatic of a loss of functionality in the photosynthetic membranes where PSII takes place. The detrimental effects of drought stress were alleviated on maize subject to 35 °C, with few differences in *ChlF* parameters between the two varieties.

The present study would indicate that heat stress has a more pronounced effect on PSII function than drought (Table 1; Figure 2, Figure 3 and Figure 4); consistent with previous field-based observations of the impact of above 40 °C temperatures when combined with drought [16,33,48] and leaf gas exchange analysis (Figure 1). The decrease in *G*_s_ (Figure 1) would reduce heat loss due to transpiration, resulting in higher leaf temperatures, which may contribute to damage to the thylakoid membranes as PSII photochemistry is reduced [18,49]. The OJIP transient suggested that impairment of PSII was more evident in drought stressed plants than in well-watered plants subject to the 35 °C treatment as more energy was dissipated per reaction center (Figure 2, Figure 3 and Figure 4). The reduced potential for photochemistry in drought plants may have worsened the impact of heat stress on the thylakoid membranes; an effect more evident in SS than in TS [17]. Reduced values of ∆V_IP_, ΨE_o_, δR_o_, and ΦR_o_ in the drought stressed sunflower plants in the 35 °C treatment suggest decreased plastoquinone A to B electron transport and PSI electron acceptors (Figure 1 and Figure 2a) [50,51]. The damage to intersystem electron transport and PSI end electron acceptor at the higher temperature likely resulted in the generation of ROS that exacerbated damage to the thylakoid membranes of drought stressed sunflower [52,53].

Heat stress has been shown to cause irreversible damage to PSII, inducing a strong rise in *F*_o_ values [14,43]; however, this was only evident in the SS variety at 35 °C, indicating greater retention of PSII functionality in well-watered TS and both maize varieties at the higher temperature (Table 1). This pattern in *F*_o_ values was generally replicated in the TBARS values of the maize and sunflower varieties (Figure 7), demonstrating the importance of membrane stability and protective mechanisms to PSII function [54,55]. In the OJIP transient, heat stress associated with growth at 35 °C produced an increase in relative variable fluorescence at 300 μs (K-band) of well-watered SS, indicative of a break-down of the oxygen evolving complex and permanent damage to the photosynthetic physiology [56].

To maintain and increase food production in hot arid regions it is necessary to identify crop varieties that tolerate drought and heat stress. Chlorophyll-fluorescence allows the rapid non-invasive screening of large numbers of crop varieties to characterize their phenotypic responses under stress conditions [34]. This compares favorably with leaf gas exchange measurements (Figure 1) that can represent a phenotyping bottle-neck [34]. Despite the potential of high throughput *ChlF* screening for categorizing phenotypic responses to drought and heat stress, few studies have investigated which *ChlF* parameters are effective in gauging the negative impacts of drought and/or heat stress on PSII as a basis for identifying tolerance. The *ChlF* measurements performed in this study are considered to be “active” and require dark-adaptation and then exposure to a saturating pulse of photosynthetically active radiation (PAR) to provide information about the capacity for photochemistry in the PSII reaction centers [14,57]. Our measurements suggest that the most effective parameter in gauging the kinetics of the drought response in both sunflower (Figure 3) and maize (Figure 4) is the *F*_m_ (Figure 2). The reduction in *F*_m_ values at lower FTSW levels indicates a reduction in the potential use of energy for photochemistry, as RubisCO carboxylation (Figure 1) [10] and the stability of photosynthetic membranes where PSI and PSII occur (Table 1; Figure 7) become impaired. The maximum quantum yield of PSII (*F*_v_/*F*_m_), ΨEo, and PI_abs_ were effective in gauging the impact of heat stress on photosynthetic performance in both species. The total number of active reaction centres per absorption (RC/ABS) parameter declined at 35 °C in the C3 sunflower and to a greater extent in the SS (Figure 3), possibly due to lower levels of photosynthetic pigments (Figure 5), carboxylation (Figure 1), and antioxidant activity (Figure 6) in the sensitive variety, resulting in less functional reaction centers in the absorbance of light energy for photochemistry. Differences in photosynthetic pigment concentrations were not so pronounced between the TM and SM varieties at 35 °C, possibly accounting for the lack of difference in RC/ABS values at 25 and 35 °C in the maize plants. The carotenoids responsible for heat dissipation as non-photochemical quenching [58] were generally higher in SS and SM (Figure 5), suggesting that the abundance of carotenoids was not a key factor in the retention of PSII photochemistry in the sensitive varieties [59].

### 3.2. Antioxidant Enzyme Responses to Drought and Heat Stress

Plant responses to a combination of stresses are different to those induced by the individual stresses [13,16,35,60,61]. Stomatal regulation is one of the most important adaptation mechanisms to environmental changes such as water deficit [10,22,32,62,63]. However, the limitation of CO_2_ availability for photosynthesis due to stomatal closure results in an increase in un-utilized light energy (Figure 1) [18,24,25,64]. This creates an imbalance between the generation and the utilization of electrons by PSII (Figure 2) and PSI (Figure 1) [20,65]. These excess electrons cause the formation of ROS in plant sub-cellular compartments under stress conditions [66,67]. The formation and antioxidant scavenging of ROS also differs under conditions of a single stress or combined multiple stresses [35,41,68]. These mechanisms are unclear in the literature, especially when comparing C3 and C4 plant species, and may account for the differences in the antioxidant responses of sunflower and maize recorded in this study. C4 metabolism has been suggested to serve as a mechanism for the avoidance of ROS production [60,68]. C4 species such as maize exhibit greater water use efficiency (WUE) than C3 species and can maintain *P*_N_ during drought and heat stress to a greater extent [10,17]. This reduces the generation of ROS, possibly accounting for the lower levels of antioxidant activity and less pronounced varietal differences observed in maize in this study. At 25 °C, the TS and SS varieties of sunflower exhibited largely similar responses to soil drying in terms of antioxidant activity (Figure 6) and lipid membrane peroxidation (Figure 7). However, at 35 °C antioxidant activity of SS declined over the duration of the experiment, possibly indicating that the protective mechanisms were being overwhelmed in the drought-sensitive variety. In contrast, antioxidant activities generally rose over the course of the study in the TS. This may explain higher levels of TBARS in the drought stressed SS at 35 °C alongside an associated increase in levels of malondialdehyde (MDA) within the plant. Glutathione reductase (GR) uses NADPH as a reductant to reduce GSSG (oxidized glutathione) to GSH (reduced glutathione), to allow binding of GSH with hydrogen peroxide (H_2_O_2_) [69,70]. Superoxide dismutase (SOD) also reduces cytotoxic O_2_^-^ to form H_2_O_2_. Hydrogen peroxide is then converted to water and oxygen by catalase (CAT) in peroxisomes, peroxidase (POX) in the chloroplast, and ascorbate peroxidase (APX) in the cytosol and chloroplast [25,70]. The activity of SOD was greater in the TS than SS at 35 °C (Figure 6), indicative of a greater capacity to neutralize O_2_^−^ [25]. Catalase activity was also greater in the TS, consistent with greater capacity to convert H_2_O_2_ produced by GR and SOD into water and oxygen [69]. In particular, POX and APX, which act within the chloroplast envelope [25,70] where the thylakoid membranes are located [29], were respectively 2.5 and 5.5 times higher in the TS than in SS (Figure 6). This may account for the greater retention of PSII electron transport of TS exposed to drought at the higher temperature (Table 1; Figure 3). A drought-tolerant C3 cotton variety also exhibited increased POX activity in comparison to a drought-sensitive variety, but no differences were recorded in APX activity between the contrasting cotton varieties [41], suggesting that antioxidant characteristics, and how these manifest as drought tolerance, vary between C3 species. The results of this study suggest that antioxidant capabilities play a more pronounced role in determining the PSII performance of C3 rather than C4 species; as the C4 photosynthetic system minimizes the production of ROS [60,68,71]. Nevertheless, antioxidants do play an important role in the prevention of membrane lipid peroxidation in maize as evidenced by the activities of CAT, APX, POX, and GR being increased under drought stress in TM at 25 and 35 °C; whereas this consistent up-regulation of antioxidant activities during drought stress was not present in SM (Figure 6). As such, the selection of drought- and heat-tolerant varieties on the basis of antioxidant capabilities (possibly associated with the retention of PSII function) is likely to be most beneficial to C3 species such as sunflower, but is also a strong factor in determining varietal tolerance to heat and drought stress in C4 species such as maize.

## 4. Materials and Methods

### 4.1. Plant Growth Conditions and Drought Application

The C4 maize (*Zea mays* L.) and C3 sunflower (*Helianthus annuus* L.) varieties were classified as drought-tolerant or -sensitive according to observations of the effect of water deficit on yield in field trials and a previous controlled environment study [10]. Seeds of the drought-sensitive (SS) (var. 08 TR 003) and -tolerant (TS) (var. Bosfora) varieties of sunflower were supplied by the Trakya Agricultural Research Institute, Edirne, Turkey. Seeds of the drought-sensitive (SM) (var. ADA-523) and -tolerant (TM) (var. ADA-9516) varieties of maize were provided by the Sakarya Maize Research Institute, Sakarya, Turkey. A plant growth room with a day/night temperature of 28/24 °C and 800 μmol m^−2^ s^−1^ photosynthetically active radiation (PAR) was used to germinate seeds sown in trays of sand supplied with a commercially available nutrient solution (COMPO Concime Universale, COMPO Italia, *Cesano Maderno*, Italy). Two weeks after germination, the seedlings were potted into nine liter square pots containing a 90% fine-sand to 10% commercial compost mixture. The pots were then placed into two controlled environment chambers which regulated light, temperature, CO_2_ (both chambers maintained ambient atmospheric (CO_2_) of 400 ppm), and humidity (both chambers maintained a relative humidity of 50%) (technical specifications of the growth chambers are given in [72]). To facilitate nutrient availability at free access rates, the plants were provided with a commercial liquid plant fertilizer (COMPO Concime Universale: NPK 7-5-7, B, Cu, Fe, Mn, Mo, Zn) once a week. The plants were watered to pot capacity every two days. 16 h of daylight (14 h at full PAR levels of 1000 μmol·m^−2^ s^−1^ with two one-hour periods of simulated dawn/dusk where light intensity was incrementally increased/decreased) were maintained in each growth chamber. Temperature regimes differed between the two controlled environment chambers: Firstly, a day/night time temperature regime of 25/20 °C (referred to as the 25 °C treatment); and secondly, the other chamber operated a day/night temperature of 35/30 °C (referred to as the 35 °C treatment). The fluctuations in temperature tracked those of PAR (including a one-hour ramping period at dawn/dusk). To elude chamber effects, the growth rooms were switched each week—no significant differences were observed in measurements performed under identical conditions in different growth chambers. To allow acclimation to the temperature regimes in the chambers, plants were grown for two weeks in the respective growth chambers. Drought stress was then imposed for 21 to 24 days.

To gauge the drought kinetics to soil drying of the drought-tolerant and -sensitive sunflower and maize, the fraction of transpirable soil water (FTSW) method [73] was used. Prior to the imposition of drought, all one hundred and ninety-two plants were watered to pot capacity. The pots were drained during the night, weighed the following morning, and then sealed in plastic bags to prevent evaporation from the soil. To ensure that anoxic conditions did not develop in the soil, the plastic bags were opened each day for approximately 10 min—this enabled the exchange of gases between the soil and the atmosphere in the growth chamber. The *G*_s_ of the plants was recorded and considered to reflect 100% potential *G*_s_ at t0. Stomatal conductance was measured using a using a PP-Systems Ciras-2 plant photosynthesis system attached to a PLC6(U) leaf cuvette and LED light unit (PP-Systems, Amesbury, MA, USA).

A total of 96 plants (four temperature/drought treatments x two varieties x three time-sampling points x four replications for each treatment) of each species were used—half were allowed to dry and the other half were maintained at pot capacity in both chambers (25 and 35 °C). The amount of water lost through transpiration was replaced for the well-watered/control plants each day. The weight of the pot and *G*_s_ were recorded every day during the study. At the point at which *G*_s_ declined to 10% (t2) of the initial *G*_s_ level or when the pot weight remained constant for three days, we considered that all of the soil water available for transpiration had been utilized (i.e., 0% FTSW), and FTSW was determined following:

(1) $$\mathrm{FTSW}=\frac{\left({\mathrm{PW}}_{\mathrm{daily}}-{\mathrm{PW}}_{\mathrm{final}}\right)}{\left({\mathrm{PW}}_{\mathrm{initial}}-{\mathrm{PW}}_{\mathrm{final}}\right)}$$

where PW indicates the pot weight in grams. The soil water content (SWC) of soil contained within the pots was measured during the 10-min period when the plastic bags covering the pots were opened. The SWC was measured using a FieldScout time domain reflectometry 100 Soil Moisture Meter (Spectrum Technologies, Aurora, IL, USA) [74]. Levels of SWC were then correlated to FTSW values for comparison in Figure 3 and Figure 4.

### 4.2. Chlorophyll a Fluorescence Transient Analysis and Parameters

Chlorophyll a fluorescence transient analysis was carried out using a portable Handy-PEA (plant efficiency analyzer) fluorimeter (Hansatech, Norfolk, UK). Measurements of *ChlF* were performed every two days, alternating between treatments throughout the imposition of drought stress. The methodology was used to analyze the performance of photosystem II (the JIP-test) and the *ChlF* parameters determined from the *ChlF* induction curve (fast kinetics) of dark-adapted leaves following exposure to a saturating pulse of light (intensity >3000 μmol photons m^−2^ s^−1^, excitation light of 650 nm) [57,75].

The software program Biolyzer 4 HP v.3 (Bioenergetics Laboratory, University of Geneva, Geneva, Switzerland) was used to analyze Handy PEA signals for OJIP test parameters. The *ChlF* transient curve plotted on a logarithmic time scale represents a polyphasic pattern. These different polyphasic time steps are labeled as O (20–50 µs), J (2 ms), I (30 ms), and P (peak). The *ChlF* parameters used in this study were *F*_o_, *F*_m_, *F*_v_/*F*_m_, *Ψ*E_o_, RC/ABS, Δ_VIP_, K-band, and the performance indices (PI_abs_ and PI_tot_). Minimum fluorescence (*F*_o_) represents the dark-adapted state of the leaf where all reaction centers of PSII are opened/oxidized and there is no electron flow in the electron transport chain (ETC) by PSII. When minimum fluorescence (*F*_o_) increases, this indicates that opened/oxidized reaction centers cannot fully oxidize and still emit fluorescence. The fluorescence maximum (*F*_m_) indicates where all reaction centers (RCs) of PSII are closed/reduced and all plastoquinone (QA) reduced. The maximum quantum yield of PSII (*F*_v_/*F*_m_) which represent efficiency by which an absorbed photon will be trapped by PSII reaction centers. *F*_v_/*F*_m_ is an indicator of photoinhibition or other kind of injury caused to the PSII complexes (Rohacek et al., 2008). The efficiency of trapped excitation energy as the movement of electrons from QA into the ETC is represented by *Ψ*E_o_. The RC/ABS is the total number of active reaction center per absorption. The IP-phase is a measure of the efficiency of electron flux through PSI to reduce the final acceptors of the ETC. The polyphasic transient measurement is very sensitive to high temperatures, and the additional K-step between the F0 and FJ at approximately 300 ms is considered to reflect sensitivity to heat stress [56,57,75]. The performance index of plants (PI_abs_) represents photosynthetic performance by reflecting functionality of both photosystems II and I. The formulae describing the parameters considered in this study are defined in Supplementary Data Table S1.

### 4.3. Leaf Sampling and Assays of Antioxidant Enzyme Activities

Fully developed leaves were taken for analyses of pigments, protein, and antioxidant enzymes: The second leaf from the flag leaf was chosen for maize (if the second leaf was not of sufficient size for all analyses the third leaf was also analyzed) and the second to fourth uppermost fully expanded leaves from sunflower. Leaves were sampled, then immediately frozen in liquid nitrogen before being stored at −80 °C prior to analysis. Leaves were sampled at three stages of the drought response: After two weeks of adaptation to the 25 and 35 °C treatments when *G*_s_ values are considered to be 100% (t0); when *G*_s_ values of the plants experiencing the drought treatment had declined by 50% (t1); and the end point of the drought stress when *G*_s_ of the plants experiencing drought had decreased to 10% of the starting *G*_s_ value (t2) or pot weight remained constant for three days. Three replicate leaf samples of approximately 0.1 g of fresh weight were taken from the middle part of each leaf from four replicate plants per variety and treatment. The leaf samples were extracted at 4 °C in Na-phosphate buffer (pH 7.8) containing 1 mM ethylenediaminetetraacetic acid (EDTA) with 2% polyvinyl pyrrolidone (PVP) (*w/v*). Samples were centrifuged at 14,000 × *g* for 30 min, and supernatants were used for the determination of protein content and enzyme activity. All spectrophotometric analyses were conducted on a Ultrospec 2100 Pro (UV-visible) spectrophotometer.

The total soluble protein content of the enzyme extracts was determined according to Bradford [76] at 595 nm, using bovine serum albumin (BSA) as a standard. Superoxide dismutase (SOD) (EC 1.15.1.1) activity was assayed from its ability to inhibit the photochemical reduction of nitrotetrazolium blue chloride (NBT) at 560 nm [77]. The reaction mixtures were incubated for 10 min under 300 µmol m^−2^ s^−1^ of light. One unit of SOD was defined as the amount of enzyme that inhibits 50% of the NBT photo reduction. Catalase (CAT) (EC 1.11.1.6) activity was estimated using the method of Bergmeyer and Gawehn [78], which measures the initial rate of decomposition of H_2_O_2_ at 240 nm for 3 min (extinction coefficient of 39.4 mM^−1^ cm^−1^) with 1 µmol H_2_O_2_ min^−1^ considered to represent 1 unit of CAT. The activity of ascorbate peroxidase (APX) (EC 1.11.1.11) was analyzed using the method of Nakano and Asada [79], where a decrease in absorbance at 290 nm and an extinction co-efficient of 2.8 mM^−1^ cm^−1^ for ascorbic acid is used to determine the oxidation of ascorbate. Determination of peroxidase (POX) (EC 1.11.1.7) activity was undertaken using the method of Kanner and Kinsella [80]. A unit of POX activity was defined as the decomposition per µmol H_2_O_2_ min^−1^ at 470 nm using an extinction coefficient of 26.6 mM^−1^ cm^−1^ for guaiacol. Glutathione reductase (GR) (EC 1.6.4.2) activity was measured according to Foyer and Halliwell [69]; where the oxidation of NADPH was measured at 340 nm, and GR activity was calculated using the extinction coefficient of NADPH (6.22 mM^−1^ cm^−1^). One unit of GR was defined as 1 µmol glutathione disulfide/oxidized glutathione (GSSG) reduced min^−1^.

### 4.4. Determination of Lipid Peroxidation

Oxidative damage to cellular membranes was gauged in terms of lipid peroxidation in the leaf samples. The accumulation of malondialdehyde (MDA) resulting from lipid peroxidation was determined by interference with thiobarbituric acid reactive substances (TBARS) measured at 532 and 600 nm according to the method of Rao and Sresty [81]. The concentration of MDA was calculated using an extinction coefficient of 155 mM^−1^ cm^−1^.

### 4.5. Determination of Pigment Contents

The foliar content of chlorophyll *a*, chlorophyll *b*, total chlorophyll, and total carotenoid pigments were determined following the method of Arnon [82] using 80% acetone. Calculations were performed using the formulae of Lichtenthaler and Buschmann [83].

### 4.6. Statistical Analyses

Statistical analyses were undertaken using SPSS 20 (IBM, New York, NY, USA). The Shapiro–Wilk test was used to assess normality and the Levene test was utilized to assess the homogeneity of variance of the *ChlF* data. The *ChlF* data were found to not be normally distributed, and the variance was not homogenous. The non-parametric Kruskal–Wallis H Test was therefore used to assess rank-based differences of means between control and treatment groups of the *ChlF* data. A three-way ANOVA was used to determine interaction effects between three factors (variety, temperature, and water status) on measured antioxidant enzymes (Table 2). A one-way ANOVA with an LSD post-hoc test was used to assess differences in variance between treatments for all other data (see Supplementary Data Table S2). A 0.05 significance level (*p* ≤ 0.05) was applied.

## 5. Conclusions

Drought events and heat waves frequently occur together, causing widespread damage to crops. Few studies have assessed varietal differences of C3 and C4 crops in antioxidant capabilities and PSII performance during drought and heat stress. Analysis of PSII electron transport using *ChlF* indicated that drought and heat stress had a greater impact in C3 sunflower than in C4 maize (Figure 3 and Figure 4); a result consistent with gas exchange analysis of CO_2_ assimilation in the same varieties (Figure 1). The TS retained PSII performance to a greater extent during drought stress at 35 °C due to enhanced antioxidant activities (Figure 6); in particular, POX and APX, which function inside the chloroplast envelope where PSII occurs. The level of antioxidant activity in the SS declined during drought stress at the higher temperature, possibly contributing to the greater impairment of PSII in SS (Figure 2a). Moreover, the TS exhibited increases in photosynthetic pigments over the duration of the study (Figure 5), indicative of a greater use of energy in photochemistry and protective dissipation of excess energy, reducing the production of harmful ROS that may have overwhelmed the protective antioxidant systems in the SS. Phenotyping on the basis of POX and APX activities alongside pigment content may be effective in developing drought- and heat-tolerant sunflower genotypes. Varietal differences in antioxidant activities were less pronounced in maize; yet, TM did consistently increase antioxidant activities during drought stress at both temperatures, whereas the SM did not respond in the same manner. This may indicate that while antioxidants play an important role in both crops, selection on the basis of enhanced antioxidant function would have a more beneficial effect in the development of drought- and heat-tolerant sunflower genotypes. Active *ChlF* was highly effective in screening the sunflower and maize varieties during drought and heat stress. Indeed, the *F*_m_ was the most sensitive parameter in discerning varietal effects of drought and heat stress in both species (Figure 2). Automated active *ChlF* analysis of the *F*_m_ may be an effective identifier of heat- and drought-tolerant varieties for more detailed analysis of leaf gas exchange and/or antioxidant capacities.

## Figures and Tables

**Table 1:** Chlorophyll fluorescence (*ChlF*) parameters of drought-tolerant and -sensitive varieties of C3 sunflower and C4 maize subject to drought and heat stress at t2. Mean values represent the average of the last two days of the experiment (eight measurements in total, therefore *n* = 4 with two technical replicates per replicate plant). Symbols indicate significant difference of the means of the treatments in comparison to the well-watered (WW) maize and sunflower varieties at 25 °C, where: ▲ indicates a significant difference to WW-TM; o indicates a significant difference to WW-SM; □ indicates a significant difference to WW-TS; and ● indicates a significant difference to WW-SS. These significant differences were assessed by the rank-based differences of the means between the control and treatment groups using the Kruskal–Wallis nonparametric test. A significance level *p* ≤ 0.05 was applied. Definitions for parameters can be found in Supplementary Data Table S1.

Treatments			*F*_o_	*F*_m_	*F*_v_/*F*_m_	*Ψ*E_o_	RC/ABS	PI_abs_	PI_tot_	I-P_phase_	K_Band_
Temp.	Var.	Water									
25 °C	**TM**	**WW**	263.4 ± 13.1	1366.1 ± 59.7○	0.775 ± 0.01	0.610 ± 0.03	4.404 ± 0.38	24.437 ± 5.71	16.001 ± 7.16	0.234 ± 0.06	0.443 ± 0.03
	TM	D	287.6 ± 24.7	1323.0 ± 75.6▲	0.746 ± 0.02○	0.600 ± 0.02	4.025 ± 0.42	18.951 ± 7.30	13.086 ± 4.04	0.248 ± 0.03○	0.467 ± 0.03
	**SM**	**WW**	280.1 ± 10.1	1455.4 ± 63.8○	0.773 ± 0.01	0.602 ± 0.01	4.221 ± 0.20	21.844 ± 2.37	11.887 ± 1.37	0.210 ± 0.00	0.458 ± 0.01
	SM	D	302.8 ± 13.7▲○	1322.9 ± 59.0○	0.728 ± 0.02▲○	0.561 ± 0.04▲○	3.569 ± 0.30▲○	12.909 ± 4.88▲○	9.171 ± 2.49○	0.234 ± 0.02	0.512 ± 0.02▲○
35 °C	TM	WW	258.3 ± 13.5○	1219.3 ± 59.7▲○	0.755 ± 0.00▲○	0.577 ± 0.02	4.267 ± 0.06	19.581 ± 2.28	14.274 ± 3.56	0.229 ± 0.02	0.445 ± 0.00
	TM	D	276.0 ± 15.0	1261.2 ± 54.9▲○	0.749 ± 0.02▲○	0.640 ± 0.01○	3.943 ± 0.31▲	21.678 ± 5.22	13.402 ± 3.54	0.240 ± 0.02	0.477 ± 0.02
	SM	WW	276.4 ± 7.3	1224.3 ± 42.3▲○	0.735 ± 0.00▲○	0.537 ± 0.02	3.961 ± 0.13	14.958 ± 2.02▲	12.216 ± 4.40	0.233 ± 0.04	0.469 ± 0.01
	SM	D	292.9 ± 25.7▲	1250.3 ± 70.8▲○	0.726 ± 0.01▲○	0.577 ± 0.03	3.646 ± 0.24▲○	14.338 ± 1.21▲○	10.465 ± 2.58	0.244 ± 0.05	0.501 ± 0.02▲○
25 °C	**TS**	**WW**	226.5 ± 9.0	1650.4 ± 40.3●	0.847 ± 0.00	0.749 ± 0.01●	6.477 ± 0.25	108.032 ± 9.44●	98.229 ± 12.29	0.355 ± 0.02	0.328 ± 0.01
	TS	D	236.4 ± 6.9	1716.0 ± 58.2□	0.845 ± 0.00	0.724 ± 0.02	6.185 ± 0.32	93.674 ± 13.73	74.735 ± 18.56	0.308 ± 0.03	0.343 ± 0.01
	**SS**	**WW**	232.5 ± 13.5	1707.3 ± 61.7□	0.846 ± 0.00	0.718 ± 0.01□	6.335 ± 0.25	89.967 ± 14.86□	82.747 ± 9.75	0.345 ± 0.01	0.334 ± 0.01
	SS	D	231.6 ± 10.9	1649.6 ± 61.3●	0.842 ± 0.00	0.723 ± 0.03	6.117 ± 0.29	89.016 ± 22.01	73.243 ± 30.65	0.316 ± 0.05	0.345 ± 0.01
35 °C	TS	WW	236.6 ± 18.4	1534.6 ± 100.3□●	0.828 ± 0.00□●	0.717 ± 0.01□	6.151 ± 0.46	75.968 ± 12.30□	73.436 ± 16.29□	0.350 ± 0.02	0.338 ± 0.02
	TS	D	261.4 ± 17.9□●	1537.1 ± 102.5□●	0.811 ± 0.00□●	0.708 ± 0.01□	5.669 ± 0.47□●	59.649 ± 11.68□●	65.109 ± 19.35□●	0.366 ± 0.02	0.360 ± 0.02□
	SS	WW	292.2 ± 38.9□●	1529.2 ± 61.2□●	0.775 ± 0.02□●	0.593 ± 0.03□●	4.548 ± 0.58□●	26.128 ± 10.52□●	33.084 ± 8.93□●	0.340 ± 0.02	0.432 ± 0.04□●
	SS	D	296.6 ± 35.0□●	1511.5 ±80.9□●	0.773 ± 0.01□●	0.555 ± 0.02□●	4.791 ±0.41□●	20.994 ±3.58□●	28.027 ±4.22□●	0.317 ± 0.02□●	0.405 ± 0.02□●

**Table 2:** Effect of drought and heat stress on chlorophyll fluorescence (*ChlF*), antioxidant, and lipid peroxidation parameters at the end of the experiment (t2) using a three-way ANOVA test. Symbols; *: *p* < 0.05, **: *p* < 0.01, ***: *p* < 0.001 represent significance levels of stress factors and their interactions on parameters; antioxidant enzymes, chlorophyll, and carotenoid content of maize and sunflower for each species independently.

Species	Factor	CAT	APX	POX	GR	SOD	TBARS	Chl *a*	Chl *b*	Tot. Chl	Tot. Carot.
**Maize**	Variety (V)	n.s.	n.s.	n.s.	**	n.s.	***	**	n.s.	*	n.s.
	Temperature (T)	***	***	***	n.s.	**	***	n.s.	n.s.	n.s.	n.s.
	Water (W)	n.s.	***	n.s.	***	**	***	n.s.	n.s.	n.s.	***
	V * T	***	*	n.s.	n.s.	n.s.	n.s.	***	***	***	n.s.
	V * W	***	n.s.	**	n.s.	n.s.	***	n.s.	***	n.s.	n.s.
	T * W	***	***	*	n.s.	***	*	**	***	n.s.	n.s.
	V * T * W	***	**	***	n.s.	n.s.	n.s.	**	*	**	n.s.
**Sunflower**	Variety (V)	***	n.s.	***	***	***	***	***	***	**	***
	Temperature (T)	n.s.	***	**	n.s.	***	***	***	***	***	***
	Water (W)	n.s.	***	**	***	*	***	n.s.	*	n.s.	***
	V * T	***	***	**	n.s.	***	***	***	***	***	**
	V * W	***	**	***	*	***	***	n.s.	***	n.s.	*
	T * W	***	***	***	n.s.	**	***	n.s.	***	n.s.	n.s.
	V * T * W	n.s.	***	***	*	***	n.s.	***	**	***	**

## Supplementary Materials

The following are available online at https://www.mdpi.com/1422-0067/21/14/4846/s1, Figure S1: FTSW response of complete ChlF parameters of sunflower, Figure S2: FTSW response of complete ChlF parameters of maize, Table S1: definitions of ChlF parameters, Table S2: ANOVA LSD *post-hoc* test results.

## Abbreviations

FTSW	Fraction of transpirable soil water
SWC	Soil water content
*P*_N_	Net photosynthesis
*G*_s_	Stomatal conductance
PSI	Photosystem *I*
PSII	Primary photochemistry/photosystem II (PSII)
*F*_v_/*F*_m_*(*φ_P0_)	The maximum quantum yield of primary photochemistry (PSII), ((*F*_m_-*F*_o_)/*F*_m_)
PAR	Photosynthetically active radiation
*Chl*	Chlorophyll
*ChlF*	Chlorophyll fluorescence
*F*_o_	Minimal fluorescence
*F_M_*	Maximal fluorescence
*F*_V_	Maximal variable fluorescence
*ψ*_E0_	The efficiency of trapped energy to move an electron further than Q_A_^−^
TBARS	Thiobarbituric acid reactive substances
SOD	Superoxide dismutase
CAT	Catalase
APX	Ascorbate peroxidases
POX	Peroxidases
GR	Glutathione reductase
TS	Drought-tolerant sunflower variety (var. Bosfora)
SS	Drought-sensitive sunflower variety (var. 08 TR 003)
TM	Drought-tolerant maize variety (var. ADA-9516)
SM	Drought-sensitive maize variety (var. ADA-523).
