# Supplementary material for: Lipid Peroxidation and Chlorophyll Fluorescence of Photosystem II Performance during Drought and Heat Stress is Associated with the Antioxidant Capacities of C3 Sunflower and C4 Maize Varieties
Source: Int J Mol Sci. 2020 Jul 9;21(14):4846. doi: 10.3390/ijms21144846 (PMC7402356; doi:10.3390/ijms21144846)
Supplement: Supplementary file 1 [file ijms-21-04846-s001.zip › Supplementary Data Table S1.pdf]

Killi et al. **“**Lipid peroxidation and chlorophyll fluorescence of photosystem II performance during drought and heat stress is associated with the antioxidant capacities of C3 sunflower and C4 maize varieties”

Supplementary Information S1.

Table S1 Chlorophyll *a* fluorescence parameters used in the fluorescence transient analysis

Technical fluorescence parameters	
**F_t**	Fluorescence emission from a dark-adapted leaf at the time t
**F_0**	Minimal fluorescence from a dark-adapted leaf
**F_M**	Maximal fluorescence from a dark-adapted leaf
**F_J**	Fluorescence intensity at the J-step (at 2 ms)
**F_I**	Fluorescence intensity at the I-step (at 30 ms)
**F_V**	Maximal variable fluorescence from a dark-adapted leaf. $F_V = F_M – F_0$
**V_J**	Relative variable fluorescence at 3ms. $V_J = (F_{2ms} – F_0) / (F_M-F_0)$
**V_I**	Relative variable fluorescence at 30ms. $V_I = (F_{30ms} – F_0) / (F_M-F_0)$
**M_0**	Slope of the curve at the origin of the fluorescence rise. It is a measure of the rate of the primary photochemistry. $M_0= 4(F_{300\mu s}-F_0) / (F_M-F_0)$
Derived parameters	
***F_v/F_m* =**	$[F_m\text{-} F_0]/F_m= \varphi_{Po} = TR_0/ABS$ = maximum quantum yield of PSII primary photochemistry, measured in samples in dark-adapted state. F_v/F_m expresses the probability that an absorbed photon will be trapped by the PSII reaction centre.
***Ψ_{Eo}* =**	$ET_0/TR_0 = 1\text{-} V_J = 1\text{-} (F_{2\ ms}\text{-}F_0)/ (F_m\ \text{-}F_0)$. Ψ_{Eo} expresses the probability that the energy of a trapped excitation is used for electron transport beyond Q_A. V_J represents the relative variable fluorescence at 2 ms (step-J) ($V_J= (F_J\text{-}F_o)/(F_m\text{-}F_o)$).
***$\Delta V_{I\text{-}P}$* =**	$1\text{-}V_I = (F_m\text{-}\ F_{30\ ms})/ (F_m\text{-}F_0)$, I-P phase (Oukarroum et al., 2009). This parameter indicates relative contribution of the I-P phase to the fluorescence transient OJIP; it is regarded as a measure of the efficiency of electron flux through PSI to reduce the final acceptors of the electron transport chain, i.e. ferredoxin and NADP. V_I indicates the relative variable fluorescence at 30 ms (step-I) ($V_I= (F_I\text{-}F_o)/(F_m\text{-}F_o)$).
**RC/ABS=**	$(1\text{-}(F_o/F_m))/(M_o/V_J)= \varphi_{Po}\ (V_J/M_0)$. This parameter represents the total number of active reaction center per absorbtion. $Mo=4(F_{300\ \mu s} – F_o)/(F_m\text{-}\ F_o)$. M_0 represents the initial slope of the double normalised fluorescence induction curve, and is a proxy of the net rate of PSII closure.
**PI_{abs} =**	(RC/ABS) $[\varphi_{Po}\ /(1\text{-}\ \varphi_{Po})]\ [\Psi E_o/(1\text{-}\ \Psi E_o)]$. Performance index on absorption bases; absorption of antenna Chls of PSII. This measure incorporates photochemical and non-photochemical processes, such as absorption and trapping of excitation energy, electron transport beyond the primary plastoquinone (QA) and dissipation of excess excitation energy.
**$PI_{tot=}$**	Performance Index total (PI_{tot}) is the potential for energy conservation from photons absorbed by PSII to the reduction flux (RE) of PSI end acceptors. The Pi_{tot} is a multi-parametric indicator of four measures of photosynthetic electron transport: (1) the concentration of reaction centres; (2) the quantum yield of PSII photochemistry; (3) the capacity for uptake of electrons in the electron chain between PSII and PSI; (4) the efficiency with which an electron can transfer from the reduced intersystem electron acceptors to the PSI end electron (Strasser et al., 2010, 2004; Tsimilli-Michael and Strasser, 2008).<br>$PI_{TOT} = PI_{ABS}\ [\delta_{Ro}/(1\text{-}\ \delta_{Ro})]$<br>where $\delta_{Ro} = (1\text{-}\ V_J)/\ (1\text{-}\ V_J) = (F_m\text{-}F_I)/\ (F_m\text{-}F_J)$.<br>$\delta_{Ro}$ is the efficiency of an electron can transport from a reduced PQ to PSI end electron acceptor.

**K-band=**	$V_{OJ}300 = (F_{300\ \mu s} - F_o)/(F_J - F_o)$. K band indicate relative variable fluorescence at 300 µs (transient normalized between F_o and F_K). This parameter express the breakdown of the oxygen-evolving system (Srivastava et al., 1997).
**ChlF steps=**	The ChlF induction phase has different time steps called as: 20–50 µs (O-step), 2 ms (J-step), 30 ms (I-step), around 0.8 s (P-step; peak) and generally denoted F_O, F_J and F_I. The last step (P-step) indicates the highest fluorescence intensity (F_m), when saturating light is used.
