# Supplementary material for: Lipid Peroxidation and Chlorophyll Fluorescence of Photosystem II Performance during Drought and Heat Stress is Associated with the Antioxidant Capacities of C3 Sunflower and C4 Maize Varieties
Source: Int J Mol Sci. 2020 Jul 9;21(14):4846. doi: 10.3390/ijms21144846 (PMC7402356; doi:10.3390/ijms21144846)
Supplement: Supplementary file 1 [file ijms-21-04846-s001.zip › Supplementary Data Table S2.pdf]

Killi et al. "Lipid peroxidation and chlorophyll fluorescence of photosystem II performance during drought and heat stress is associated with the antioxidant capacities of C3 sunflower and C4 maize varieties"

Supplementary Information S2.

Table S2; <u>LSD *Post-Hoc* Test Results:</u>

Analysis performed; maize and sunflower seperatly for each antioxidant enzymes,

Maize (8 treatments) within the all sampling point.

Sunflower (8 treatments) within the all sampling point.

Treatments	CAT								
	t0			t1			t2		
1C-TM	0.0054	±0.0006	defgh	0.0052	±0.0005	defgh	0.0041	±0.0003	ghi
1D-TM	0.0054	±0.0006	defgh	0.0029	±0.0003	ikl	0.0051	±0.0006	defgh
1C-SM	0.0069	±0.0005	cd	0.0096	±0.0007	b	0.0121	±0.0014	a
1D-SM	0.0069	±0.0005	cd	0.0049	±0.0006	efgh	0.0043	±0.0007	fghi
2C-TM	0.0064	±0.0007	cde	0.0056	±0.0005	defg	0.0040	±0.0005	ghi
2D-TM	0.0061	±0.0006	cdef	0.0045	±0.0005	efghi	0.0078	±0.0004	c
2C-SM	0.0035	±0.0003	hik	0.0044	±0.0008	fghi	0.0016	±0.0002	l
2D-SM	0.0036	±0.0003	ghik	0.0021	±0.0003	kl	0.0043	±0.0004	fghi
1C-TS	0.0009	±0.0000	b	0.0008	±0.0000	cd	0.0015	±0.0000	ef
1D-TS	0.0184	±0.0009	b	0.0040	±0.0007	ij	0.0030	±0.0005	ijk
1C-SS	0.0226	±0.0022	a	0.0146	±0.0020	c	0.0056	±0.0006	hi
1D-SS	0.0192	±0.0014	b	0.0121	±0.0006	cde	0.0056	±0.0006	hi
2C-TS	0.0006	±0.0000	hij	0.0006	±0.0000	gh	0.0013	±0.0000	def
2D-TS	0.0006	±0.0000	hij	0.0004	±0.0000	ijk	0.0007	±0.0000	fg
2C-SS	0.0008	±0.0000	k	0.0029	±0.0005	ijk	0.0041	±0.0004	ij
2D-SS	0.0008	±0.0000	k	0.0024	±0.0003	jk	0.0032	±0.0003	ijk

	APX								
Treatments	t0			t1			t2		
1C-TM	0.664	±0.09	def	0.534	±0.07	def	0.525	±0.13	def
1D-TM	0.664	±0.09	def	0.572	±0.03	def	0.862	±0.15	d
1C-SM	0.400	±0.10	ef	0.499	±0.05	def	0.554	±0.02	def
1D-SM	0.400	±0.10	ef	0.478	±0.05	def	0.413	±0.08	ef
2C-TM	0.504	±0.04	def	0.442	±0.03	def	0.381	±0.09	f
2D-TM	0.504	±0.04	def	0.838	±0.28	de	1.324	±0.14	c
2C-SM	2.567	±0.20	a	1.451	±0.09	c	0.337	±0.02	f
2D-SM	2.567	±0.20	a	0.634	±0.01	def	2.078	±0.31	b
1C-TS	2.881	±0.09	a	1.677	±0.07	bcd	0.611	±0.07	fg
1D-TS	2.881	±0.09	a	0.650	±0.06	fg	0.189	±0.01	g
1C-SS	2.087	±0.59	bc	1.352	±0.33	cdef	0.617	±0.08	fg
1D-SS	2.087	±0.59	bc	2.304	±0.13	ab	1.282	±0.31	def
2C-TS	0.563	±0.07	fg	0.653	±0.09	fg	0.905	±0.05	efg
2D-TS	0.563	±0.07	fg	1.520	±0.50	bcde	1.510	±0.17	bcde
2C-SS	0.673	±0.08	fg	0.643	±0.03	fg	0.613	±0.08	fg
2D-SS	0.673	±0.08	fg	0.637	±0.13	fg	0.744	±0.07	fg

	POX								
Treatments	t0			t1			t2		
1C-TM	0.200	±0.00	b	0.061	±0.00	bc	0.108	±0.00	b
1D-TM	0.726	±0.20	b	0.579	±0.12	bcde	1.089	±0.29	a
1C-SM	0.279	±0.03	def	0.694	±0.09	b	1.295	±0.05	a
1D-SM	0.279	±0.03	def	0.514	±0.08	bcdef	0.382	±0.09	bcdef
2C-TM	0.029	±0.00	bcd	0.036	±0.00	bcdef	0.057	±0.00	bcdef
2D-TM	0.029	±0.00	bcd	0.191	±0.00	bcd	0.159	±0.00	bc
2C-SM	0.228	±0.01	ef	0.310	±0.01	cdef	0.355	±0.04	bcdef
2D-SM	0.198	±0.03	f	0.270	±0.03	def	0.615	±0.11	bcd
1C-TS	1.934	±0.20	ab	1.977	±0.24	a	1.566	±0.03	abc
1D-TS	1.934	±0.20	ab	0.475	±0.07	fgh	0.315	±0.06	gh
1C-SS	1.280	±0.38	cde	0.920	±0.21	ef	0.560	±0.07	fgh
1D-SS	1.280	±0.38	cde	1.471	±0.15	bcd	0.606	±0.18	fgh
2C-TS	0.514	±0.06	fgh	0.825	±0.06	efg	1.136	±0.14	cde
2D-TS	0.514	±0.06	fgh	0.967	±0.04	def	1.136	±0.14	cde
2C-SS	0.205	±0.02	h	0.309	±0.02	gh	0.452	±0.05	fgh
2D-SS	0.205	±0.02	h	0.235	±0.05	h	0.235	±0.01	h

	GR								
**Treatments**	t0			t1			t2		
1C-TM	0.237	±0.04	bc	0.162	±0.02	bcdefg	0.106	±0.00	defg
1D-TM	0.360	±0.12	a	0.087	±0.01	efg	0.164	±0.01	bcdefg
1C-SM	0.085	±0.01	efg	0.119	±0.01	cdefg	0.152	±0.03	bcdefg
1D-SM	0.116	±0.02	ab	0.051	±0.01	g	0.270	±0.04	cdefg
2C-TM	0.148	±0.01	bcdefg	0.126	±0.01	cdefg	0.068	±0.01	fg
2D-TM	0.235	±0.02	bcd	0.183	±0.03	bcdef	0.213	±0.01	bcde
2C-SM	0.272	±0.06	ab	0.189	±0.02	bcdef	0.106	±0.02	defg
2D-SM	0.180	±0.02	ab	0.137	±0.04	cdefg	0.273	±0.02	bcdefg
1C-TS	0.261	±0.01	a	0.196	±0.02	bc	0.155	±0.02	cd
1D-TS	0.261	±0.01	a	0.038	±0.00	g	0.047	±0.01	fg
1C-SS	0.121	±0.01	de	0.090	±0.01	ef	0.052	±0.00	fg
1D-SS	0.121	±0.01	de	0.215	±0.02	b	0.200	±0.03	bc
2C-TS	0.060	±0.00	fg	0.061	±0.00	fg	0.062	±0.00	fg
2D-TS	0.060	±0.00	fg	0.056	±0.01	fg	0.159	±0.02	cd
2C-SS	0.038	±0.00	g	0.056	±0.00	fg	0.093	±0.00	ef
2D-SS	0.038	±0.00	g	0.036	±0.01	g	0.036	±0.00	g

	SOD								
**Treatments**	t0			t1			t2		
1C-TM	88.6	±10.1	b	69.9	±4.6	bcd	51.2	±4.6	def
1D-TM	88.6	±10.1	b	40.7	±5.6	f	45.6	±4.5	f
1C-SM	82.6	±3.4	bc	69.7	±3.3	bcd	56.8	±5.0	def
1D-SM	82.6	±3.4	bc	45.4	±4.5	f	41.1	±4.9	f
2C-TM	87.3	±7.7	b	64.4	±3.8	ef	41.6	±1.6	cde
2D-TM	87.3	±7.7	b	46.4	±5.1	f	64.7	±4.3	cde
2C-SM	114.1	±8.4	a	80.1	±3.7	bc	46.2	±3.6	ef
2D-SM	114.1	±8.4	a	67.4	±7.2	cd	79.5	±6.5	bc
1C-TS	71.6	±4.8	b	68.4	±1.4	b	65.2	±5.2	bc
1D-TS	71.6	±4.8	b	58.5	±5.3	bc	21.8	±1.7	e
1C-SS	66.0	±6.3	bc	51.1	±3.4	cd	36.1	±3.7	de
1D-SS	66.0	±6.3	bc	112.8	±7.0	a	51.2	±4.7	cd
2C-TS	42.3	±6.0	d	71.9	±4.5	b	101.4	±5.1	a
2D-TS	42.3	±6.1	d	38.8	±4.6	d	58.0	±4.2	bc
2C-SS	21.6	±1.7	e	36.4	±3.5	de	51.1	±6.2	cd
2D-SS	21.6	±1.7	e	22.9	±3.1	e	40.7	±4.2	d

	TBARS								
Treatments	t0			t1			t2		
1C-TM	33.5	±2.0	ef	48.0	±1.8	d	62.4	±2.4	c
1D-TM	33.5	±2.0	ef	75.4	±2.1	b	103.5	±4.0	a
1C-SM	33.9	±1.0	ef	45.9	±2.1	d	58.0	±4.5	c
1D-SM	33.9	±1.0	ef	41.3	±4.0	de	77.6	±5.1	b
2C-TM	31.9	±3.2	f	27.5	±2.0	fg	23.0	±1.7	gh
2D-TM	31.9	±3.2	f	44.0	±5.2	d	56.5	±5.0	c
2C-SM	13.7	±1.4	i	18.8	±1.7	hi	23.8	±3.0	gh
2D-SM	13.7	±1.4	i	76.6	±2.0	b	28.2	±1.6	fg
1C-TS	24.5	±1.0	ghij	28.0	±1.2	defghi	31.5	±1.7	defg
1D-TS	25.5	±1.1	ghij	34.5	±2.6	d	65.6	±8.3	a
1C-SS	25.4	±1.0	ghij	27.2	±0.8	efghi	29.1	±2.0	defgh
1D-SS	23.8	±0.9	hij	26.2	±4.2	ghij	57.8	±3.5	b
2C-TS	9.0	±0.6	k	21.3	±1.4	ij	33.6	±3.0	def
2D-TS	9.0	±0.6	k	19.5	±1.7	i	41.0	±1.7	c
2C-SS	9.4	±0.8	k	21.6	±0.6	ij	33.8	±1.4	de
2D-SS	9.4	±0.8	k	26.8	±1.6	fghi	46.3	±2.2	c

Treatments	Chl.a								
	t0			t1			t2		
1C-TM	1.108	±0.05	hi	1.530	±0.16	def	1.833	±0.23	bc
1D-TM	1.108	±0.05	hi	2.273	±0.12	a	1.334	±0.11	efgh
1C-SM	1.654	±0.05	cd	1.560	±0.05	de	1.117	±0.02	hi
1D-SM	1.654	±0.05	cd	1.493	±0.02	def	1.043	±0.07	i
2C-TM	0.762	±0.00	j	1.029	±0.08	i	1.163	±0.00	ghi
2D-TM	0.762	±0.00	j	1.895	±0.10	bc	1.477	±0.09	def
2C-SM	1.257	±0.00	fghi	1.348	±0.02	efgh	1.440	±0.04	def
2D-SM	1.257	±0.00	fghi	1.986	±0.08	b	1.395	±0.01	defg
1C-TS	1.049	±0.02	de	1.316	±0.01	b	1.597	±0.01	a
1D-TS	1.049	±0.02	de	1.116	±0.01	cd	1.636	±0.10	a
1C-SS	1.118	±0.04	cd	1.221	±0.01	bc	1.335	±0.03	b
1D-SS	1.118	±0.04	cd	0.927	±0.03	efg	1.605	±0.06	a
2C-TS	0.653	±0.06	i	0.772	±0.04	ghi	0.890	±0.03	efgh
2D-TS	0.653	±0.06	i	0.809	±0.05	fgh	0.764	±0.03	hi
2C-SS	1.034	±0.04	de	0.942	±0.04	ef	0.849	±0.06	fgh
2D-SS	1.034	±0.04	de	0.941	±0.00	ef	0.622	±0.02	i

	Chl.b								
Treatments	t0			t1			t2		
1C-TM	0.283	±0.01	defg	0.343	±0.03	bcd	0.346	±0.06	bcd
1D-TM	0.283	±0.01	defg	0.404	±0.02	ab	0.266	±0.01	fg
1C-SM	0.425	±0.01	a	0.228	±0.01	g	0.119	±0.00	h
1D-SM	0.425	±0.01	a	0.323	±0.00	efg	0.273	±0.02	cdef
2C-TM	0.164	±0.00	h	0.246	±0.01	g	0.264	±0.00	fg
2D-TM	0.164	±0.00	h	0.377	±0.03	abc	0.140	±0.00	h
2C-SM	0.315	±0.00	cdef	0.325	±0.00	cdef	0.335	±0.00	cde
2D-SM	0.315	±0.00	cdef	0.403	±0.01	ab	0.283	±0.01	defg
1C-TS	0.290	±0.00	defg	0.334	±0.00	bcd	0.377	±0.00	b
1D-TS	0.290	±0.00	defg	0.350	±0.00	bc	0.525	±0.04	a
1C-SS	0.317	±0.01	cde	0.324	±0.00	cde	0.328	±0.01	bcd
1D-SS	0.317	±0.01	cde	0.315	±0.01	cde	0.550	±0.03	a
2C-TS	0.239	±0.01	g	0.260	±0.01	fg	0.282	±0.01	defg
2D-TS	0.239	±0.01	g	0.273	±0.01	efg	0.246	±0.00	g
2C-SS	0.336	±0.01	bcd	0.320	±0.01	cde	0.304	±0.01	cdef
2D-SS	0.336	±0.01	bcd	0.321	±0.00	cde	0.254	±0.00	fg

	Tot.Chl								
Treatments	t0			t1			t2		
1C-TM	1.391	±0.06	efgh	1.873	±0.20	cd	2.179	±0.29	b
1D-TM	1.391	±0.06	efgh	2.677	±0.14	a	1.599	±0.12	defg
1C-SM	2.078	±0.06	bc	1.788	±0.05	cd	1.236	±0.02	h
1D-SM	2.078	±0.06	bc	1.817	±0.02	cd	1.317	±0.07	fgh
2C-TM	0.927	±0.01	i	1.275	±0.10	gh	1.426	±0.00	efgh
2D-TM	0.927	±0.01	i	2.272	±0.10	b	1.617	±0.10	def
2C-SM	1.572	±0.00	defg	1.673	±0.02	de	1.774	±0.05	cd
2D-SM	1.572	±0.00	defg	2.389	±0.09	b	1.678	±0.01	de
1C-TS	1.340	±0.03	cdefg	1.650	±0.01	b	1.974	±0.01	a
1D-TS	1.340	±0.03	cdefg	1.466	±0.01	bcd	2.160	±0.19	a
1C-SS	1.434	±0.06	cde	1.545	±0.02	bc	1.662	±0.04	b
1D-SS	1.434	±0.06	cde	1.241	±0.05	efgh	2.155	±0.08	a
2C-TS	0.892	±0.08	jk	1.032	±0.05	ijk	1.172	±0.04	fghi
2D-TS	0.892	±0.08	jk	1.082	±0.06	hij	1.011	±0.03	ijk
2C-SS	1.370	±0.05	cdef	1.261	±0.06	defgh	1.153	±0.08	ghi
2D-SS	1.370	±0.05	cdef	1.261	±0.00	defgh	0.876	±0.02	k

	Tot.Carotenoid								
Treatments	t0			t1			t2		
1C-TM	0.180	±0.02	cdefg	0.186	±0.02	bcdefg	0.193	±0.03	abcdef
1D-TM	0.180	±0.02	cdefg	0.236	±0.03	abc	0.236	±0.02	ab
1C-SM	0.217	±0.00	abcde	0.208	±0.00	abcdef	0.170	±0.00	efghi
1D-SM	0.217	±0.00	abcde	0.227	±0.02	abcd	0.220	±0.02	abcde
2C-TM	0.126	±0.00	hi	0.137	±0.00	ghi	0.121	±0.00	i
2D-TM	0.126	±0.00	hi	0.239	±0.02	ab	0.242	±0.01	a
2C-SM	0.177	±0.00	defgh	0.155	±0.00	fghi	0.156	±0.00	fghi
2D-SM	0.177	±0.00	defgh	0.243	±0.00	a	0.210	±0.01	abcde
1C-TS	0.110	±0.00	defg	0.125	±0.00	d	0.153	±0.00	c
1D-TS	0.110	±0.00	defg	0.121	±0.00	d	0.196	±0.01	b
1C-SS	0.106	±0.00	defgh	0.109	±0.00	defg	0.113	±0.00	def
1D-SS	0.106	±0.00	defgh	0.118	±0.00	de	0.220	±0.00	a
2C-TS	0.082	±0.00	i	0.085	±0.00	ij	0.087	±0.00	ij
2D-TS	0.082	±0.00	i	0.090	±0.00	hij	0.091	±0.00	ghij
2C-SS	0.107	±0.00	defg	0.102	±0.00	efghi	0.097	±0.00	fghij
2D-SS	0.107	±0.00	defg	0.109	±0.00	defg	0.124	±0.00	d

Description of the Treatments:

**1:** 25 °C	
**2:** 35 °C	
**TM:** Tolerant Maize	
**SM:** Sensitive Maize	
**TS:** Tolerant Sunflower	
**SS:** Sensitive Sunflower	
**C:** Control/ well-watered	
**D:** Drought	
