# Supplementary figures and images for: Lipid Peroxidation and Chlorophyll Fluorescence of Photosystem II Performance during Drought and Heat Stress is Associated with the Antioxidant Capacities of C3 Sunflower and C4 Maize Varieties
Source: Int J Mol Sci. 2020 Jul 9;21(14):4846. doi: 10.3390/ijms21144846 (PMC7402356; doi:10.3390/ijms21144846)

# Supplementary Data S1

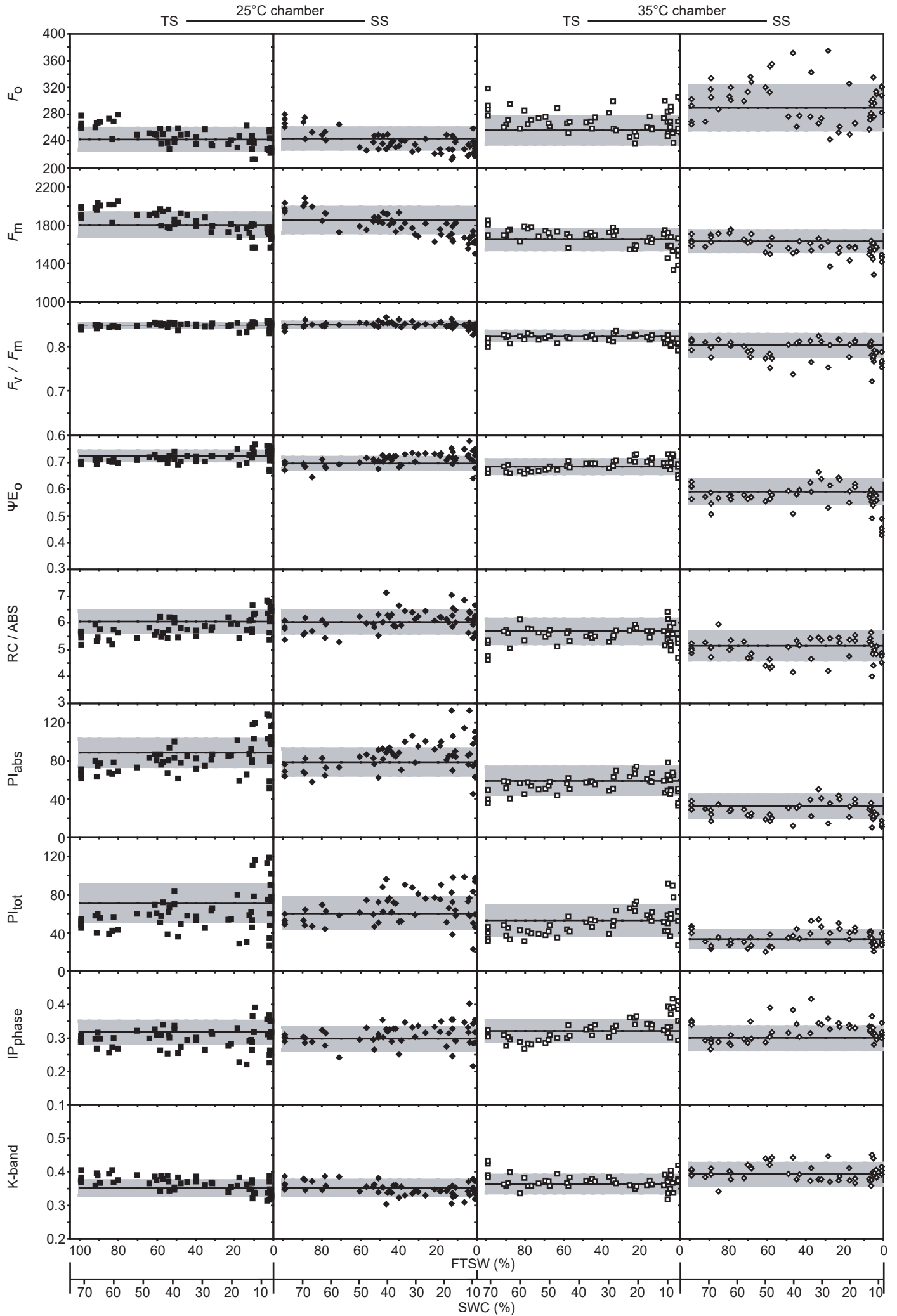

Supplement: Supplementary file 1 [file ijms-21-04846-s001.zip › Supplementary data Figure S1.pdf]

# Supplementary Data S2

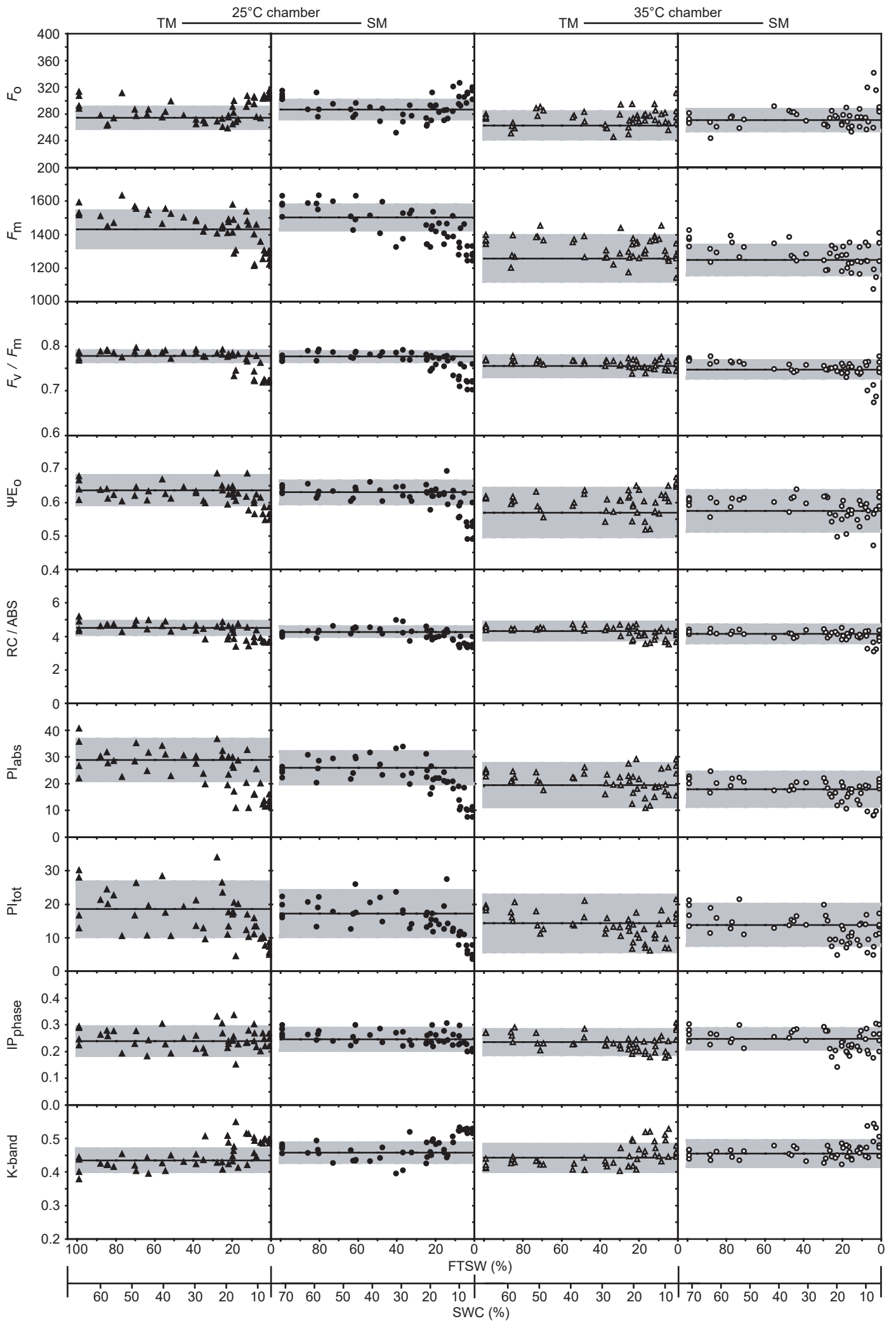

Supplement: Supplementary file 1 [file ijms-21-04846-s001.zip › Supplementary data Figure S2.pdf]
